# Probing the Internal pH and Permeability of a Carboxysome Shell

**DOI:** 10.1021/acs.biomac.2c00781

**Published:** 2022-09-02

**Authors:** Jiafeng Huang, Qiuyao Jiang, Mengru Yang, Gregory F. Dykes, Samantha L. Weetman, Wei Xin, Hai-Lun He, Lu-Ning Liu

**Affiliations:** † Institute of Systems, Molecular and Integrative Biology, University of Liverpool, Crown Street, Liverpool L69 7ZB, United Kingdom; ‡ School of Life Sciences, Central South University, Changsha 410017, China; § Department of Central Laboratory, Shandong Provincial Hospital Affiliated to Shandong First Medical University, Jinan 250021, China; ∥ Medical Science and Technology Innovation Center, Shandong First Medical University & Shandong Academy of Medical Sciences, Jinan 271000, China; ⊥ College of Marine Life Sciences, and Frontiers Science Center for Deep Ocean Multispheres and Earth System, Ocean University of China, Qingdao 266003, China

## Abstract

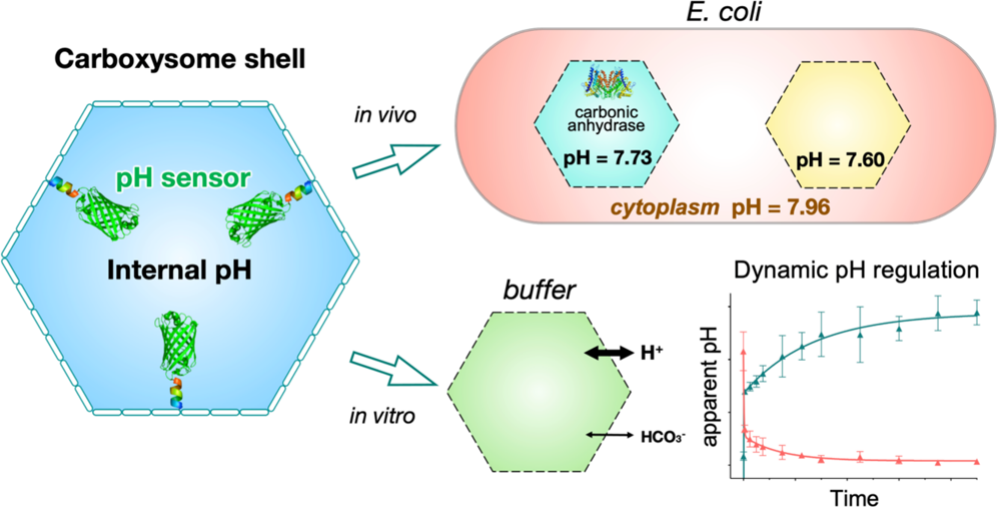

The carboxysome is a protein-based nanoscale organelle in cyanobacteria and many proteobacteria, which encapsulates the key CO_2_-fixing enzymes ribulose-1,5-bisphosphate carboxylase/oxygenase (Rubisco) and carbonic anhydrase (CA) within a polyhedral protein shell. The intrinsic self-assembly and architectural features of carboxysomes and the semipermeability of the protein shell provide the foundation for the accumulation of CO_2_ within carboxysomes and enhanced carboxylation. Here, we develop an approach to determine the interior pH conditions and inorganic carbon accumulation within an α-carboxysome shell derived from a chemoautotrophic proteobacterium *Halothiobacillus neapolitanus* and evaluate the shell permeability. By incorporating a pH reporter, pHluorin2, within empty α-carboxysome shells produced in *Escherichia coli*, we probe the interior pH of the protein shells with and without CA. Our *in vivo* and *in vitro* results demonstrate a lower interior pH of α-carboxysome shells than the cytoplasmic pH and buffer pH, as well as the modulation of the interior pH in response to changes in external environments, indicating the shell permeability to bicarbonate ions and protons. We further determine the saturated HCO_3_^–^ concentration of 15 mM within α-carboxysome shells and show the CA-mediated increase in the interior CO_2_ level. Uncovering the interior physiochemical microenvironment of carboxysomes is crucial for understanding the mechanisms underlying carboxysomal shell permeability and enhancement of Rubisco carboxylation within carboxysomes. Such fundamental knowledge may inform reprogramming carboxysomes to improve metabolism and recruit foreign enzymes for enhanced catalytical performance.

## 1 Introduction

In cells, multiple proteins can self-assemble to form a variety of supercomplex structures and organelles, compartmentalizing biochemical reactions and protecting enzymes/molecules within specific microenvironments from the highly dynamic and challenging cellular conditions.^1−6^ Among them, nanoscale protein “cages” play central roles in chemical storage, nucleic acid packaging, cargo delivery, and cellular metabolism.^7−9^ The majority of protein cages in nature are formed through precise, dense protein packing and adopt diverse mechanisms of permeability to allow and control the passage of substrates and products. Given their self-assembly, encapsulation, and permeability features, along with their high biocompatibility compared with chemical materials, protein cages have attracted increasing attention from academics and industrials to design and engineer new nanocontainers and scaffolding biomaterials for catalytic enhancement, enzyme stabilization, and molecule delivery.^10,11^

**Figure 1:**
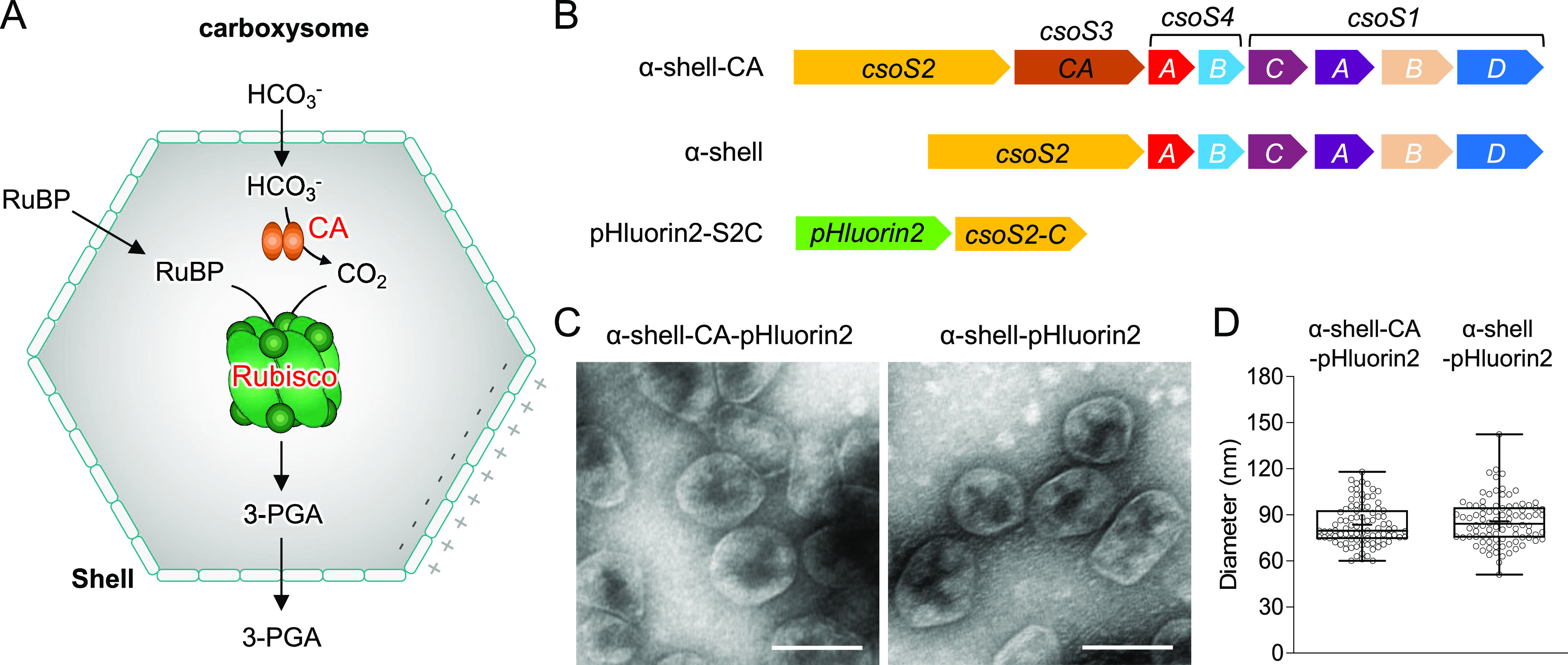
Expression and characterization of α-carboxysome shells encapsulating pH-sensitive pHluorin2. (A) Schematic of the carboxysome structure and metabolic pathways. The carboxysome shell serves as a physical barrier for controlling the flux of specific metabolites in and out of the carboxysome. The shell permits the passage of bicarbonate (HCO_3_^–^) and ribulose-1,5-bisphosphate (RuBP) into the carboxysome. Carbonic anhydrase (CA) in the carboxysome lumen dehydrates HCO_3_^–^ to CO_2_ and provides high levels of CO_2_ around Rubisco to facilitate the carboxylation of RuBP by adding CO_2_ to generate 3-phosphoglycerate (3-PGA), which is then transported across the shell and is metabolized via the Calvin–Benson–Bassham cycle. The Rubisco and CA activities as well as the directional charge properties of shell proteins play roles in determining the luminal pH. (B) Genetic organizations of the α-shell-CA (the α-carboxysome shell with encapsulated carbonic anhydrase, CA), α-shell (the α-carboxysome shell without CA), and pHluorin2-S2C (expressing free pHluorin2 fused with CsoS2 C-terminus) constructs. (C) Electron microscopy (EM) of isolated α-shell-CA-pHluorin2 (left) and α-shell-pHluorin2 (right) from the 20% sucrose fractions. Scale bar: 100 nm. (D) Diameters of purified α-shell-CA-pHluorin2 (83.55 ± 13.46 nm, *n* = 100) and α-shell-pHluorin2 (85.71 ± 14.89 nm, *n* = 100) from the 20% sucrose fractions are comparable (*p* = 0.3104).

Carboxysomes are specialized protein organelles for CO_2_ fixation in cyanobacteria and some chemoautotrophs and play an important role in the global carbon cycle and primary productivity.^12−16^ Carboxysomes encapsulate the key CO_2_-fixing enzymes ribulose-1,5-bisphosphate carboxylase-oxygenase (Rubisco) and carbonic anhydrase (CA) using a polyhedral semipermeable shell.^17^ In cyanobacteria, bicarbonate (HCO_3_^–^) is pumped from the external environment into the cell cytoplasm through membrane-spanning bicarbonate transporters and is accumulated within the cell cytoplasm.^18−22^ The carboxysome shell is selectively permeable to charged HCO_3_^–^, allowing substantial accumulation of HCO_3_^–^ within the organelle^23,24^ (Figure 1A). The co-encapsulated CA then dehydrates HCO_3_^–^ to CO_2_, optimizing the carboxysome internal pH and elevating CO_2_ levels around Rubisco.^25−27^ The inherent organizational features of the carboxysome and the semipermeability of the protein shell provide the structural foundation for overcoming the inherent low affinity of Rubisco for CO_2_ and enhancing Rubisco carboxylation.^2,13^ These naturally occurring characteristics also make the carboxysomes attractive candidates in synthetic engineering to improve CO_2_ fixation, metabolism, and growth of non-native organisms.^28−36^

The carboxysome shell serves as a physical barrier to create special internal conditions distinct from the cytoplasmic environment, and the central pores of shell proteins likely provide portals for molecule influx and efflux of the carboxysome.^23,24^ The interior pH is a critical parameter in a variety of physiochemical processes and is influenced by the concentrations of charged ions inside the protein shell. The interior pH inside the carboxysome shell is established through HCO_3_^–^ influx, steady-state chemical equilibrium between HCO_3_^–^ and CO_2_, and proton production by Rubisco carboxylation, and is crucial for the enzymatic rates of Rubisco and CA.^27,37^ A lower pH may lead to elevated CO_2_ by shifting the equilibrium from HCO_3_^–^ toward CO_2_.^25,38^ While attempts have been made to determine the carboxysomal pH and internal conditions,^25,39^ the actual internal pH and HCO_3_^–^/CO_2_ accumulation within the carboxysome have not yet been well characterized.

The α-carboxysome of the chemoautotrophic bacterium *Halothiobacillus neapolitanus* has been well studied as a model carboxysome in fundamental research and synthetic engineering. The protein components constructing α-carboxysomes in *H. neapolitanus* are encoded by a set of genes located mainly in a *cso* operon in the genome, involving the *cbbLS* genes (encoding Rubisco large and small subunits), *csoSCA* (encoding carbonic anhydrase), *csoS2* (encoding CsoS2 for cargo-shell association), *csoS4A/B* and *csoS1A/B/C* that encode shell proteins, as well as the *csoS1D* gene that is ∼11 kbp downstream of the *cso* operon and encodes pseudohexameric shell proteins.^16,40,41^ Our recent studies have demonstrated that two types of α-carboxysome shells (α-shell-CA and α-shell) can be generated by expressing the *cso* operon and *csoS1D* in *Escherichia coli*.^36^ The α-shell-CA shell contains shell proteins (CsoS2, CsoS4A/B, CsoS1C/A/B, CsoS1D) and CA, suggesting that CA can associate with empty shells without Rubisco,^36^ whereas α-shell comprises only the shell proteins without CA (Figure 1B). Both α-carboxysome shells have essentially similar shapes as native α-carboxysomes from *H. neapolitanus*.^36^

Here, we determine the carboxysome interior pH *in vivo* and *in vitro* under different external environments using the synthetic empty α-carboxysome shells and a fluorescence indicator pHluorin2, an enhanced ratiometric pH-sensitive green fluorescent protein (GFP) with a pH-dependent bimodal excitation spectrum.^42−44^ We reveal a more acidic internal pH of α-carboxysomes than the external environment and evaluate the shell permeability to protons and HCO_3_^–^ ions. This study provides insight into the physiochemical properties of the carboxysome interior created by shell permeability, which is crucial for enhanced Rubisco carboxylation in carboxysomes.

## 2 Materials and Methods

### 2.1 Generation of Constructs

The nucleotide sequence of the α-carboxysome shell operon encoding CsoS2, CsoSCA, CsoS4A, CsoS4B, CsoS1C, CsoS1A, CsoS1B, and CsoS1D was amplified from the genome of *Halothiobacillus neapolitanus* and was cloned into the pBAD vector linearized by *Nco*I and *Eco*RI (α-shell-CA), as described previously.^36^ The α-shell construct was the same as α-shell-CA except for the absence of the gene encoding CsoSCA (Figure 1A). The pHluorin2 gene was cloned from pME-pHluorin2 (Addgene #73794), fused with CsoS2 C-terminus, and inserted into a pBAD33 vector via PCR amplification (pHluorin2-S2C, Table S1). All primers used in this report are listed in Table S2. All of the expression plasmids were transformed into *E. coli* DH5α and BL21(DE3) cells. Vector expression was carried out in *E. coli* BL21(DE3) cells that were grown aerobically at 37 °C in lysogeny broth (LB) medium containing 25 μg mL^–1^ chloramphenicol and 100 μg mL^–1^ ampicillin. HiFi CloneAmp polymerase and Gibson assembly kit were purchased from New England Biolabs (U.K.). All chemicals and reagents were purchased from Sigma-Aldrich unless otherwise specified.

### 2.2 Expression and Purification of α-Shell-CA-pHluorin2 and α-Shell-pHluorinS2

*E. coli* BL21(DE3) cells containing pHluorin2-S2C and α-shell-CA (or α-shell) vectors were cultured at 37 °C until OD_600_ reaches 0.6, and then the expression of pHluorin2-S2C alone or pHluorin2-S2C and α-shell-CA (or α-shell) was induced by 1 mM arabinose at 25 °C for 16 h with constant shaking, as reported previously.^36,45^ Purification of synthetic α-carboxysome shells was carried out as described previously.^36^ The cell pellet was resuspended in a tetramethylbenzidine (TMB) buffer (5 mM Tris-HCl, 10 mM MgCl_2_, and 20 mM NaHCO_3_, pH 7.8) with 1% protease inhibitor cocktail (Thermo Fisher, U.K.), and then the cells were broken by Cell Homogenizer (Stansted Fluid Power, U.K.). Cell debris was removed by centrifugation at 10,000*g*, followed by centrifugation at 50,000*g* to enrich α-shell-CA or α-shell. The pellets were resuspended in the TMB buffer and were loaded onto a step sucrose density gradient (10–50% sucrose in TMB buffer, w/v) followed by ultracentrifugation at 105,000*g* for 30 min at 4 °C. The collected α-shell-CA or α-shell samples were further concentrated by ultracentrifugation at 105,000*g* and stored in 5 mM Tris-HCl (pH 8.0) or other buffers at 4 °C.

### 2.3 Sodium Dodecyl Sulfate Polyacrylamide Gel Electrophoresis (SDS-PAGE) and Immunoblot Analysis

SDS-PAGE and immunoblot examination were performed following the procedure described previously.^35,46−48^ A total of 30 μg proteins were loaded into each well. Immunoblot analysis was performed using primary mouse monoclonal anti-GFP (Invitrogen, 33-2600, dilution 1:2000), rabbit polyclonal anti-CsoS1 (Agrisera, Sweden, AS14 2760, dilution 1:3000), and horseradish peroxidase-conjugated goat anti-mouse IgG secondary antibody (Agrisera, Sweden, AS10 988, dilution 1:10,000) and anti-rabbit IgG secondary antibody (Agrisera, Sweden, AS09 602, dilution 1:10,000). Signals were visualized using a chemiluminescence kit (Bio-Rad). Immunoblot images were collected by ImageQuant LAS 4000 software version 1.2.1.119. Immunoblot protein quantification was performed using ImageJ (NIH Image, version 1.53c). For each experiment, at least three biological repeats were examined.

### 2.4 Transmission Electron Microscopy

Preparation of isolated shell structures for negative-staining transmission electron microscopy was performed as described earlier.^14,15,31,36,46−48^ Images were recorded using an FEI Tecnai G2 Spirit BioTWIN transmission electron microscope equipped with a Gatan Rio 16 camera. Image analysis was carried out using ImageJ (NIH Image, version 1.53c).

### 2.5 Live-Cell Confocal Fluorescence Microscopy

After the culturing of *E. coli* BL21(DE3) cells and the expression of pHluorin2-S2C alone or pHluorin2-S2C and α-shell-CA (or α-shell) induced by 1 mM arabinose at 25 °C for 16 h, the *E. coli* cells were prepared on agar plates for confocal microscopy, as described earlier.^31,49,50^ The *E. coli* cells were imaged using a Zeiss LSM780 with a 63 × oil-immersion objective with excitation wavelength at 488 nm and emission detection at 500–520 nm. Image analysis was performed using ImageJ.

### 2.6 Fluorescence Spectrophotometric Measurements

Fluorescence spectrum scanning was performed using an F2700 spectrofluorimeter (Hitachi, Japan) at room temperature. Fluorescence excitation spectra were recorded between 350 and 500 nm with the emission at 508 nm (bandwidth of 5 nm for excitation and 5 nm for emission). Each spectrum was an average of three scans from three independent biological replicates. Fluorescence time-lapse scanning was determined from 0 to 1500 s.

### 2.7 Intracellular pH Assays

**Figure 2:**
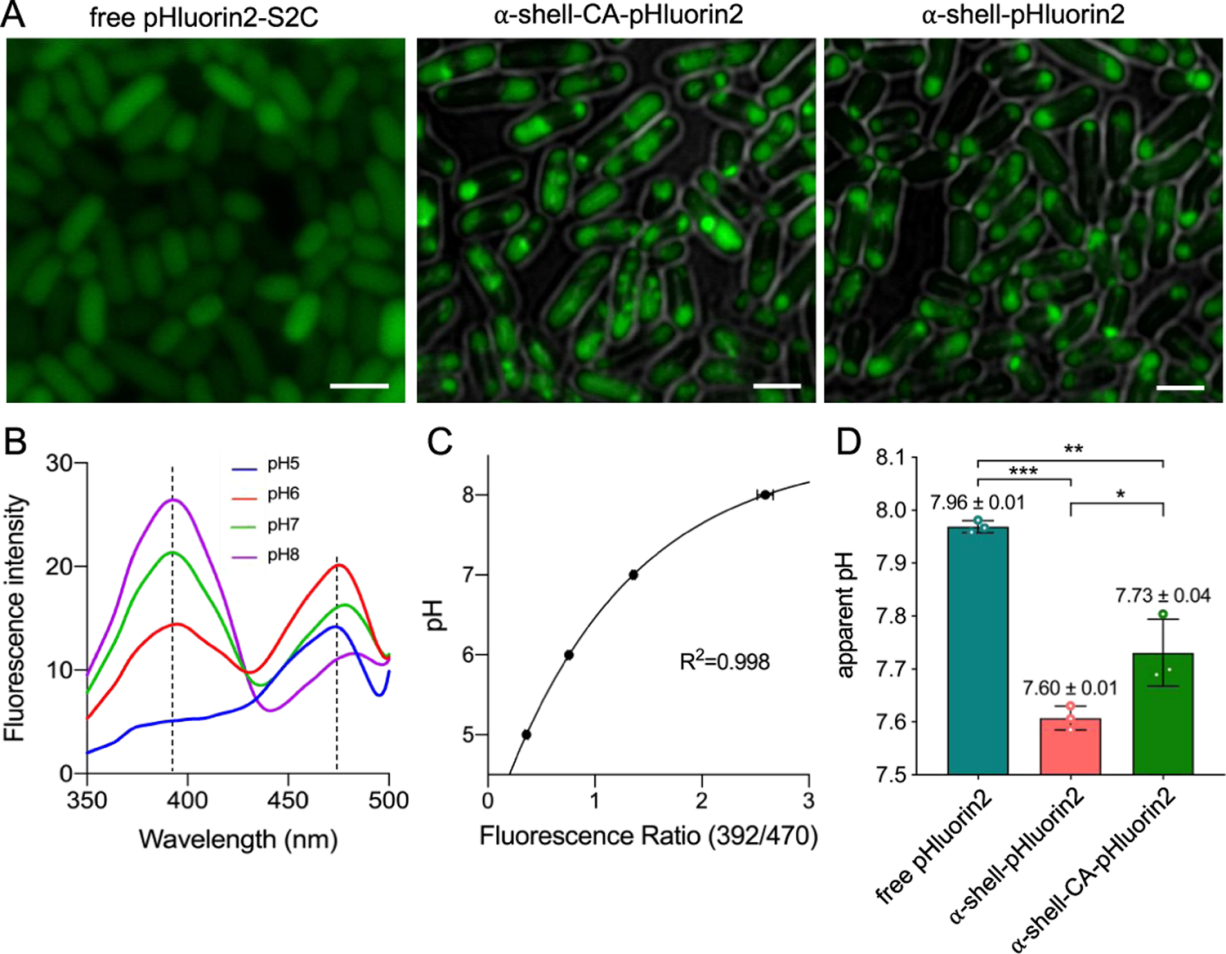
Determination of the cytosolic and α-carboxysome shell internal pH in *E. coli*. (A) Fluorescence microscopy images of exponentially growing *E. coli* cells expressing free pHluorin2, α-shell-pHluorin2, and α-shell-CA-pHluorin2, indicating that pHluorin2 fused with CsoS2 C-terminus was encapsulated in the formed synthetic α-carboxysome shell assemblies, α-shell, and α-shell-CA. Scale bar: 2 μm. (B) Fluorescence excitation spectra of free pHluorin2 in *E. coli* at pH 5–8 with the emission at 508 nm show two fluorescence peaks at 392 and 470 nm. (C) Calibration curve of free pHluorin2 in *E. coli* treated with sodium benzoate at the medium pH varying between 4.5 and 8.5. Sodium benzoate treatment resulted in the disruption of the permeability and proton motive force of the cell membrane to ensure equilibration between the cytoplasmic pH (reflected by free pHluorin2 fluorescence) and the extracellular pH. (D) Determination of *in situ* cytoplasmic pH and the internal pH of α-shell and α-shell-CA in *E. coli* reveals that the internal pH of both α-shell and α-shell-CA was significantly lower than the cytosolic pH in *E. coli* (*p* = 0.0029 for α-shell vs free pHluorin2, *p* = 0.0158 for α-shell-CA vs free pHluorin2, *p* = 0.036 for α-shell vs α-shell-CA). The results are representative of three independent experiments (Figure S3), analyzed by one-way analysis of variance (ANOVA). *, *p* < 0.05; **, *p* < 0.01.

*E. coli* BL21(DE3) cells containing the pHluorin2-S2C vector, pHluorin2-S2C vector with α-shell-CA (or α-shell) vectors were co-expressed as described above. The cells were pelleted by centrifugation at 1000*g* and resuspended to *A*_600_ = 1.0 with buffers at the pH ranging from 5.0 to 9.0. Citrate buffer (0.1 M, for pH 5.0 and 6.0) and Tris-HCl buffer (0.1 M, for pH 7.0 and 8.0) were used in this study. Sodium benzoate was added to a final concentration of 40 mM at each pH (for proton motive force disruption). After 20 min incubation, the ratios of fluorescence excitation at 392 and 470 nm of pHluorin2 in cells at the corresponding pH were measured to establish a calibration curve using the Boltzmann best-fitting equation (Figure 2B,C). The cytoplasmic pH and interior pH of α-shell and α-shell-CA in *E. coli* cells were measured based on the calibration curve.

### 2.8 *In Vitro* pH Assays

The ratios of fluorescence excitation at 392 and 470 nm of isolated α-shell-CA-pHluorin2 and α-shell-pHluorin2 synthetic shells in the TMB buffer were measured. The calibration curve was plotted by the ratios of fluorescence excitation at 392 and 470 nm of free pHluorin2-S2C at different pH (0.1 M citrate buffer for pH 5.0 and 6.0; 0.1 M Tris-HCl buffer for pH 7.0, 7.5, 8.0 and 8.5), fitted by the Boltzmann best-fitting equation. The interior pH of α-shell and α-shell-CA in solutions were measured based on the established calibration curve.

### 2.9 HCO_3_^–^ Diffusion Assays

For HCO_3_^–^ diffusion assays, α-shell-pHluorin2 was treated with 20 mM NaHCO_3_ in ddH_2_O (pH 8.6, measured by pH meter) for 1 h, followed by centrifugation at 50,000*g* to enrich α-shell-pHluorin2. The pellets were resuspended in ddH_2_O without NaHCO_3_ (pH 7.5, measured by pH meter). For the HCO_3_^–^ absorption assays, α-shell-pHluorin2 was incubated in ddH_2_O (pH 7.5) for 1 h, followed by centrifugation at 50,000*g* to enrich α-shell-pHluorin2. The pellets were resuspended in 20 mM NaHCO_3_ dissolved in ddH_2_O (pH 8.6). To elucidate the effects of HCO_3_^–^ on the shell internal environment, changes in shell internal pH as a function of HCO_3_^–^ concentration were examined. The α-shell-pHluorin2 samples were incubated in ddH_2_O for 1 h, followed by centrifugation at 50,000*g* to enrich α-shell-pHluorin2. The pellets were resuspended in different concentrations of NaHCO_3_ for 1 h. Fluorescence spectrum scanning was performed using an F2700 spectrofluorimeter until the pH value reached an equilibrium level.

### 2.10 Statistical Analysis

Statistical evaluation was performed using GraphPad Prism 7.0a (GraphPad Software). Statistical significance of differences between means of pH measured using each assay was tested using one-way analysis of variance (ANOVA). To confirm a statistically significant difference between the mean values of two groups, the Student’s *t*-test was applied. A *p* value of < 0.05 was considered to indicate a significant difference between two groups. For the derivate values, the combined standard error formula was used to calculate the standard deviations (SD). All values were presented as mean ± SD and were plotted as the mean values with SD.

## 3 Results and Discussion

### 3.1 Development of a System to Probe the Interior pH within the α-Carboxysome Shell

To probe the internal pH environment of the α-carboxysome shells, we use an enhanced pH-sensitive fluorescent protein, pHluorin2, as a pH indicator. The local pH environment can be indicated by the ratio of fluorescence excitation at 392 and 470 nm of the ratiometric pHluorin2,^51^ which has been applied to determine the cytoplasmic, vacuolar, and extracellular pH of cells.^44,52^ The C-terminus of CosS2 (S2C) can serve as an encapsulation peptide to recruit external proteins into the α-carboxysome shell.^36^ Therefore, we fused the gene encoding pHluorin2 to the 5′ end of the gene fragment encoding CsoS2 C-terminus (pHluorin2-S2C) in a pBAD33 vector (Figure 1B) and co-expressed pHluorin2-S2C with α-carboxysome shells in *E. coli*, to obtain both synthetic α-shell-CA and α-shell shells encapsulating pHluorin2.

Sucrose gradient centrifugation and sodium dodecyl sulfate polyacrylamide gel electrophoresis (SDS-PAGE) combined with immunoblot analysis using anti-GFP antibodies and anti-CsoS1A/C antibodies showed the enrichment of pHluorin2-S2C and the major shell proteins in the 20–30% sucrose fractions, with relatively higher levels in the 20% fraction (Figure S1). In contrast, free pHluorin2-S2C proteins without shell encapsulation were predominantly distributed in the supernatant on top of the sucrose gradient (Figure S1). These results indicated the encapsulation of pHluorin2-S2C into the α-carboxysome shells. Negative-staining electron microscopy (EM) of the 20% sucrose fraction revealed that the recombinant shells containing pHluorin2-S2C exhibit a polyhedral shape, with a diameter of 83.6 ± 13.5 nm (*n* = 100) for α-shell-CA encapsulating pHluorin2 (α-shell-CA-pHluorin2) and 85.7 ± 14.9 nm (*n* = 100) for α-shell encapsulating pHluorin2 (α-shell-pHluorin2) (Figure 1C,D), both morphologically indistinguishable from the empty shells as reported previously.^36^ The EM results confirmed that pHluorin2-S2C encapsulation has no remarkable effects on shell assembly and architecture.

### 3.2 Shells Create a Lower-pH Microenvironment from the Cytoplasm

Live-cell confocal fluorescence imaging showed that when pHluorin2-S2C was co-expressed with the α-carboxysome shells, the spotty fluorescence signal was visualized in *E. coli* (Figure 2A), consistent with the previously observed clustered shell structures in *E. coli* using confocal fluorescence microscopy and thin-section electron microscopy.^36^ In contrast, pHluorin2 fluorescence was distributed throughout the *E. coli* cytoplasm in the absence of shells when expressing pHluorin2 alone in *E. coli*. These results, together with EM of isolated shells (Figure 1C), confirmed that expression of α-shell-CA and α-shell resulted in the formation of polyhedral shells in *E. coli* and efficient encapsulation of pHluorin2-S2C within the α-shell-CA and α-shell. Notably, the formed shell structures tend to form large clusters in *E. coli* cells, probably due to the absence of the cognate McdAB system (Maintenance of Carboxysome Distribution protein A and B) from *H. neapolitanus*, which has been shown to be essential for determining the proper positioning of carboxysomes in cells.^53^

The *E. coli* strain that expresses free pHluorin2 was used to probe the intracellular pH out of synthetic α-carboxysome shells within the *E. coli* cell. To establish *in situ* calibration curves for pH measurements, *E. coli* cultures were grown to an OD_600_ of 0.6 before live-cell confocal imaging and were subjected to treatment with sodium benzoate, an antibacterial reagent that can disrupt the permeability and proton motive force of the cell membrane to ensure equilibration between the cytoplasmic pH and the extracellular pH.^51,54^ After incubation with 40 mM sodium benzoate, the *E. coli* cells expressing free pHluorin2 were incubated in buffers at the pH range of 5.0–8.0 (pH 5.0 and 6.0 using 0.1 M citrate buffer, pH 7.0 and 8.0 using 0.1 M Tris-HCl buffer). The gradual decline in the fluorescence excitation at 392 nm and the increase in fluorescence at 470 nm were observed with the decrease in the external pH (Figure 2B). The ratios of pHluorin2 fluorescence at 392 and 470 nm were plotted against different pH and were fitted with the Boltzmann equation to achieve the calibration curve for *in vivo* measurements (Figures 2C and S2).

Based on the established correlation between pHluorin2 fluorescence 392/470 nm ratios and buffer pH, we determined the cytoplasmic pH of *E. coli* cells without sodium benzoate treatment. The fluorescence 392/470 nm ratio of free pHluorin2 was 2.53 ± 0.02 (*n* = 3), reflecting the cytoplasmic pH of 7.96 ± 0.01 (Figures 2D and S3), which is in good agreement with the previously estimated pH range between 7.2 and 8.0.^54−56^ With the encapsulation of shell structures, the fluorescence 392/470 nm ratios of pHluorin2 were 2.01 ± 0.02 (*n* = 3) for α-shell and 2.18 ± 0.07 (*n* = 3) for α-shell-CA, indicating that the apparent pH values of the α-shell and α-shell-CA lumen were 7.60 ± 0.01 and 7.73 ± 0.04, respectively (Figure 2D). Both pH values were lower than the *E. coli* cytosolic pH value (7.96 ± 0.01), suggesting that the intact carboxysome shell could maintain a pH gradient between interior and external environments. The results are consistent with the notion that the carboxysome shell has an acidic interior environment^57,58^ and are supported by recent mathematical modeling,^37,38,59^ although the pH gradient was not detected previously using the α-carboxysome mutant, wherein CbbS was fused with pHluorin2.^25^

The formation of an acidic environment within the carboxysome shell may be explained by a Donnan equilibrium^60^ and the natural properties of the carboxysome shell. According to the Donnan equilibrium, the protein shell separates two solutions and prevents ion species with specific charges to pass through the pores, resulting in the imbalance of ion species across the shell. Computational simulations indicated that the carboxysome shell is permeable to charged HCO_3_^–^ and ensures accumulation of HCO_3_^–^/CO_2_ within the shell.^23,24^ Moreover, the inner surface of the α-carboxysome shell appears to be largely negatively charged,^58^ which provides the foundation for attracting protons and creating a lower pH inside the shell than the external buffer pH. The pH gradient between the interior and outside environment across proteinaceous shells may play a universal role in ensuring molecule packing and enzyme function. A pH gradient and acidic interior pH may favor higher concentrations of CO_2_ within the carboxysome, by shifting the equilibrium from HCO_3_^–^ toward CO_2_, and may lead to a higher degree of Rubisco saturation and improvement of the CCM performance.^37,38^ Likewise, a lower interior pH of catabolic bacterial microcompartments in *Salmonella* is assumed to optimize the concentration of volatile metabolites.^61^ Viral capsids that are structurally analogous to bacterial microcompartments could also function as a physical barrier to proton diffusion^62,63^ and create a more acidic environment with pH ∼ 0.5 units lower than that of the outside solution.^64^ Our results further showed that α-shell-CA has a higher internal pH than α-shell (Figure 2D, *p* = 0.036), presumably due to the presence of encapsulated CA that converts HCO_3_^–^ to CO_2_ and hence promotes the establishment of an HCO_3_^–^ gradient across the shell and HCO_3_^–^ influx (see details below).

### 3.3 Modulation of the Interior pH of Isolated α-Carboxysome Shells *In Vitro*

**Figure 3:**
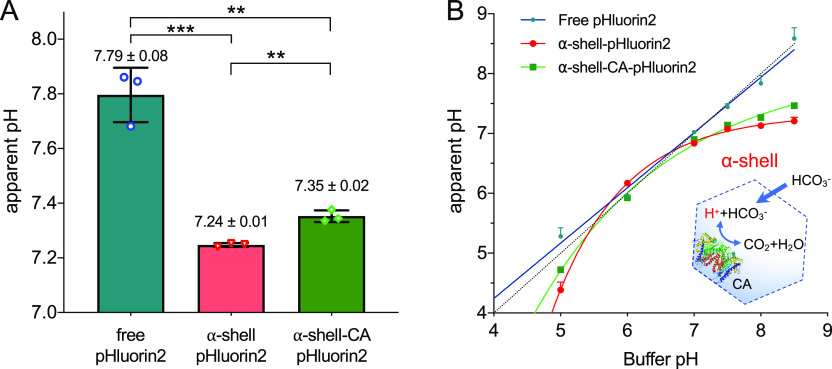
Carboxysome shell has a lower interior pH than external pH and is permeable to protons. (A) Determination of the α-carboxysome shell internal pH using isolated α-shell and α-shell-CA shells in the TMB buffer. The results show that the interior pH of α-carboxysome shells is lower than external buffer pH; in addition, the interior pH of α-shell is lower than that of α-shell-CA (*p* = 0.0095 for α-shell vs free pHluorin2, *p* = 0.0239 for α-shell-CA vs free pHluorin2, *p* = 0.0223 for α-shell vs α-shell-CA). Data are representative of three independent experiments, analyzed by one-way analysis of variance (ANOVA). *, *p* < 0.05; **, *p* < 0.01. (B) Dynamic changes in the interior pH of α-shell and α-shell-CA at varying external pH, indicating that the α-carboxysome shell is permeable to protons. The interior pH of α-shell (red line) is lower than that of α-shell-CA (green line) when the buffer pH was above 7.0 and below 5.5, likely due to the catalytic activities of CA (PDB: 2FGY), which convert HCO_3_^–^ to CO_2_ (inset). The external pH measured based on the fluorescence of free pHluorin2 was used as a control (teal line). The dashed line indicates the linear relationship between buffer pH and measured pH. Data are representative of three independent experiments.

To further characterize the internal pH of the α-carboxysome shell, α-shell-CA-pHluorin2 and α-shell-pHluorin2 were isolated using sucrose gradient ultracentrifuge in the TMB buffer (5 mM Tris-HCl, 10 mM MgCl_2_, 20 mM NaHCO_3_, pH 7.8). Fluorescence spectroscopic analysis revealed that the internal pH of the α-shell (7.24 ± 0.01, *n* = 3) was lower than that of the α-shell-CA (7.35 ± 0.02, *n* = 3), both lower than the TMB buffer pH (7.79 ± 0.08, *n* = 3) (Figure 3A).

To study the modulation of the interior pH of α-carboxysome shells at varying pH, we first established the correlation between the fluorescence 392/470 nm ratios of free pHluorin2 *in vitro* and buffer pH in the range of 5.0–8.5 (pH 5.0 and 6.0 using 100 mM citrate buffer, pH 7.0–8.5 using 100 mM Tris-HCl buffer). Then, the pHluorin2 fluorescence 392/470 nm ratios of α-shell-CA-pHluorin2 and α-shell-pHluorin2 at pH 5.0–8.5 were determined (Figures 3B and S2). The results showed that the apparent internal pH of α-shell-CA and α-shell differed as the buffer pH changed. When the buffer pH was above 7.0 and below 5.5, pHluorin2 encased in the α-shell-CA and α-shell exhibited lower fluorescence 392/470 nm ratios than free pHluorin2 (Figure 3B). This finding suggested that the α-carboxysome shell is permeable to protons, in line with previous observations,^25^ and verified a more acidic pH microenvironment within the α-carboxysome shell than external pH, consistent with our *in vivo* observations (Figure 2). Indeed, molecular simulations showed the permeability of the pores of carboxysome shell protein to negatively charged ions such as HCO_3_^–^, ribulose-1,5-bisphosphate (RuBP), and 3-phosphoglycerate (3-PGA).^23,24^ Note that, due to the limited response timescale of pHluorin2 and detection approaches as well as the absence of Rubisco, we could not determine the accurate migration rate of protons across the shell (mathematically estimated as 10^–4^ m s^–1^)^27^ in their native context and therefore, could not exclude the possibility that the carboxysome shell has certain resistance to proton migration. Our results also showed that the α-shell lacking CA has a lower internal pH than the α-shell-CA at pH > 7.5 (Figure 3B), in agreement with *in vivo* results (Figure 2D), likely owing to the presence of CA that can convert HCO_3_^–^ and protons to CO_2_ and H_2_O.

To verify whether the pH difference between the internal and external environments of α-carboxysome shells was generated by the shell barrier, we treated α-shell-CA-pHluorin2 and α-shell-pHluorin2 with trypsin and then determined the ratios of pHluorin2 fluorescence at 392/470 nm. The results revealed that disturbance of the outer shell led to the decrease in the internal pH of α-shell-CA and α-shell and eventually a pH equilibration across the α-carboxysome shell (Figure S4), demonstrating the importance of the structural integrity of synthetic shells for shell permeability.

Collectively, our results provide experimental evidence that intact α-carboxysome shells are permeable to protons and can maintain a lower internal pH from the cell cytoplasm or external buffer. In addition, the established system for determining the internal pH of α-carboxysome shells provides a means for studying the permeability characteristics of carboxysomes, which is extendable to other bacterial microcompartment shells and protein organelles.

### 3.4 Carboxysome Shell Permeability to HCO_3_^–^

Negatively charged HCO_3_^–^ is the substrate of carboxysomal CA and should diffuse across the carboxysome shell through the positively charged central pores of shell proteins for Rubisco carboxylation,^23,24^ although mathematical modeling speculated that CCM may not require selective uptake of HCO_3_^–^ into the carboxysome.^37,38^ The decreased interior pH of native carboxysomes might be due to the presence of RuBP, 3-PGA, and protons required or produced by Rubisco carboxylation within a diffusion-limited compartment.^27^ Here, using empty shells, we could evaluate the pH changes derived solely from the natural permeability of carboxysome shells to HCO_3_^–^.

**Figure 4:**
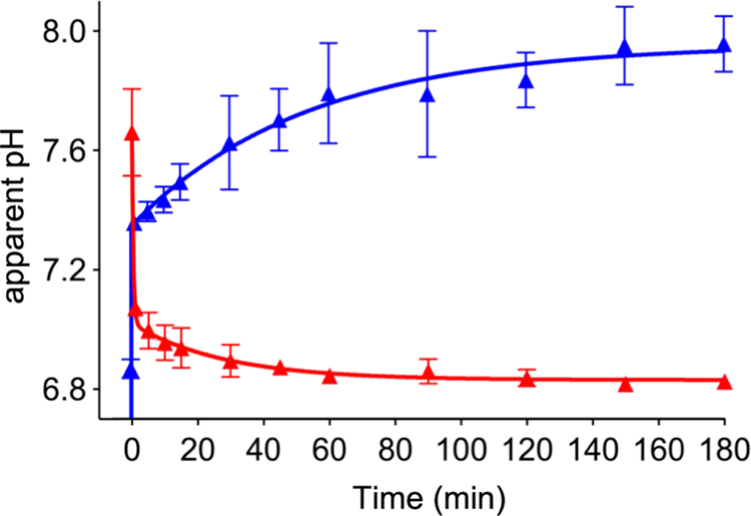
Dynamic changes in the interior pH of intact α-shells in response to rapid external HCO_3_^–^ variation by time-lapse fluorescence measurements. Red, The α-shell-pHluorin2 shells were pretreated with 20 mM NaHCO_3_ (pH 8.6) for 1 h and were resuspended in ddH_2_O (pH 7.5). The internal pH of α-shell was then estimated by measuring the fluorescence of pHluorin2 as a function of time. Blue, The α-shell-pHluorin2 shells were incubated in ddH_2_O (pH 7.5) for 1 h, followed by resuspension in 20 mM NaHCO_3_ (pH 8.6). The internal pH of α-shell was then estimated by measuring the fluorescence of pHluorin2 as a function of time. The data were fitted with a “Two-phase decay” model using GraphPad Prism. Data are representative of three independent experiments.

By detecting the changes in internal pH of α-carboxysome shells using pHluorin2 and time-lapse fluorescence assays, we experimentally accounted for the dynamics of HCO_3_^–^ passage across the empty shells to evaluate the intrinsic permeability of carboxysome shells to HCO_3_^–^. First, α-shell-pHluorin2 was incubated in ddH_2_O for 1 h to empty carried ions. It was then resuspended in 20 mM NaHCO_3_ dissolved in ddH_2_O (pH 8.6). This buffer exchange resulted in a rapid increase in the buffer pH (Figure S5). In contrast, the internal pH of α-shell increased rapidly in response to the immediate raise of external pH (Figure 4, blue, 0–1 min), implicating a fast proton efflux through the shell.^27^ Consistently, it has been shown that α-carboxysomes could quickly respond to external pH changes at the millisecond level.^25^ Then, the interior pH of the shell rose gently from 7.35 to 7.80, presumably due to the relatively moderate influx of HCO_3_^–^ into shells, and finally reached an equilibrium level of HCO_3_^–^ within the shell. We also evaluated the internal pH changes when altering the order of ddH_2_O and NaHCO_3_ treatments. α-shell-pHluorin2 was treated with 20 mM NaHCO_3_ (pH 8.6) to elevate internal NaHCO_3_ levels and then resuspended in ddH_2_O (pH 7.5). As shown in Figure 4 (red), the α-shell internal pH declined drastically (0–1 min), reflecting a fast proton influx, followed by a gentle drop, which might be ascribed to relatively moderate HCO_3_^–^ efflux.

Taken together, our results emphasize the dynamic proton permeability and HCO_3_^–^ passage across the carboxysome shell and show that the penetration rate of protons is significantly higher than that of HCO_3_^–^. This is in good agreement with mathematical modeling results,^27^ which inferred that protons have a higher migration rate of 10^–4^ m s^–1^ across the carboxysome shell, whereas the permeability of ions including HCO_3_^–^ (10^–6^ m s^–1^) is 2 orders of magnitude slower than that of protons. It is worth noting that our results represent the spectroscopic features of bulk shells enriched in buffer solutions. With the development of single-molecule imaging techniques, such as Interferometric Scattering Anti-Brownian Electrokinetic (ISABEL) trap, it may be possible to accurately determine the shell permeability to HCO_3_^–^ using the α-shell-pHluorin2 system at the single-particle level.^65^ Moreover, an effective detection method and shell variants in the presence of CA and/or Rubisco remain to be developed and investigated to measure the accurate permeability rates of ions (such as HCO_3_^–^, RuBP, and _3_^–^PGA) across the protein shell.

### 3.5 High HCO_3_^–^ Concertation Is Necessary for Acidification in the Carboxysome Shell

**Figure 5:**
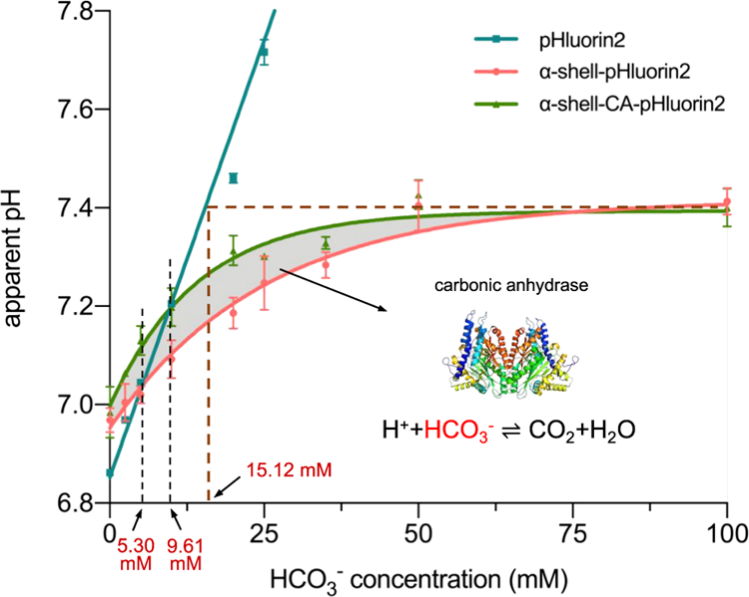
Modulation of the interior pH response of the α-carboxysome shells. Detection of the interior pH of the α-carboxysome shells as a function of external HCO_3_^–^ concentration, fitted with an one-phase association exponential equation. At the concentration of HCO_3_^–^ lower than 5.30 mM, the interior pH of α-shell (red line) was higher than the surrounding buffer pH (teal line). In the presence of CA (α-shell-CA, green line), the interior pH of α-shell-CA was higher than the external buffer pH before the concentration of HCO_3_^–^ reached 9.61 mM. The α-carboxysome shell interior pH increased gradually at 0–75 mM HCO_3_^–^, and reached the plateau above 75 mM. The maximum interior pH of α-shell and α-shell-CA implies that the saturated HCO_3_^–^ concentration within the α-carboxysome shell is 15.12 mM. The interior pH of α-shell-CA (green) is higher than that of α-shell (red) at 0–75 mM HCO_3_^–^, likely due to the catalytic activities of CA (PDB: 2FGY), which convert HCO_3_^–^ to CO_2_, resulting in a higher HCO_3_^–^ influx. The buffer pH measured based on pHluorin2 fluorescence is shown in teal. Data are representative of three independent experiments.

To further evaluate the effects of HCO_3_^–^ on the internal pH of the carboxysome shell, we determined the changes in the internal pH at different external HCO_3_^–^ concentrations after 1 h incubation (Figure 5). When the concentration of HCO_3_^–^ was lower than 5.30 mM, the interior pH of α-shell (red line) was higher than the surrounding buffer pH (teal line). In the presence of CA (α-shell-CA, green line), the interior pH of α-shell-CA was higher than the external buffer pH before the concentration of HCO_3_^–^ reached 9.61 mM. These results indicated that high HCO_3_^–^ concertation (>10 mM) is necessary for acidification in carboxysome shells. Consistently, a lower cytosolic HCO_3_^–^ concertation than 10 mM was proposed to be insufficient to saturate the carboxysomal Rubisco with CO_2_.^37^ The catalytic activity of encapsulated CA in converting rapidly HCO_3_^–^ into CO_2_ may result in the discrepancy between the internal pH of α-shell and α-shell-CA, as discussed above.

As the external HCO_3_^–^ level rose, the interior pH of both α-shell-CA and α-shell increased and reached a plateau above 75 mM HCO_3_^–^. The maximum internal pH of both α-shell and α-shell-CA is essentially the same (∼pH 7.4), implicating similar saturated HCO_3_^–^ levels within α-shell and α-shell-CA. This interior pH condition is equivalent to the buffer pH (indicated by free pHluorin2 fluorescence) at the HCO_3_^–^ level of 15.12 mM (Figure 5), suggesting that the saturated HCO_3_^–^ concentration in α-shell and α-shell-CA is ∼15 mM. Consistently, the intracellular inorganic carbon (HCO_3_^–^ and CO_2_) levels of cyanobacterial species were between 15 and 30 mM, up to 1000-fold compared to exogenous HCO_3_^–^ in low CO_2_ environments, important for driving the CCM.^26,66^

Moreover, α-shell-CA possessed a higher internal pH than α-shell at the external HCO_3_^–^ concentration of 0–75 mM (Figure 5), due to the presence of CA within the shell and distinct diffusion behaviors of HCO_3_^–^ and CO_2_ across the shell. The encapsulated CA catalyzes the reversible interconversion of CO_2_ + H_2_O to HCO_3_^–^ + H^+^, generating an HCO_3_^–^ gradient across the shell to promote HCO_3_^–^ influx and consequently resulting in elevated internal pH of α-shell-CA. A recent study has also revealed that the CA content in *E. coli* expressed α-carboxysomes was greatly reduced compared to that in native α-carboxysomes from *H. neapolitanus*.^16^ Whether the CA abundance in the carboxysome or in the engineered shells determines the levels of changes in internal pH and HCO_3_^–^ concentration merits further investigation. In addition, the higher HCO_3_^–^ influx could also lead to the rise of HCO_3_^–^/CO_2_ equilibrium concentration and eventually a higher CO_2_ within α-shell-CA. The combination of CA catalysis and a higher HCO_3_^–^/CO_2_ equilibrium concentration within α-shell-CA may facilitate accumulation of CO_2_ and Rubisco carboxylation in native carboxysomes.

CCM plays a central role in enhancing Rubisco carboxylation.^2^ In the carboxysome-containing microbes, CCM involves active accumulation of inorganic carbon in the cytosol via numerous membrane-spinning HCO_3_^–^ pumps and elevation of CO_2_ levels around Rubisco by encapsulation of Rubisco with CA within carboxysomes to improve carbon fixation.^2,22,67^ Apart from the co-condensation of Rubisco and CA that can produce protons inside the carboxysome,^27^ our results characterized the intrinsic feature of the permeability of the α-carboxysome shell to HCO_3_^–^diffusion, which could enable influx of HCO_3_^–^ and generation of a more acidic environment inside the carboxysome shell. These effects may facilitate CO_2_ accumulation and thus favor Rubisco carboxylation and photosynthetic efficiency.^37^ It is worth noting that our experiments were performed in *E. coli* or synthetic α-carboxysome shells and in the absence of cognate HCO_3_^–^ transporters and Rubisco, which might alter the pH conditions and internal proton production. The actual internal environment of native α-carboxysomes within their natural hosts remains to be explored.

## 4 Conclusions

The semipermeability of the carboxysome shell is not only fundamental for carboxysome biogenesis and carbon fixation but also a key feature that makes the carboxysome shell an attractive candidate in bioengineering of new nanobioreactors to supercharge cell metabolism of non-native hosts. Here, we experimentally measured the interior pH and permeability to bicarbonate ions of α-carboxysome shells with or without CA. We showed that the interior pH of α-shell-CA and α-shell in *E. coli* is about 7.73 and 7.60, respectively, both lower than *E. coli* cytosolic pH of 7.96; the changes in external pH could lead to the regulation of the interior pH of α-carboxysome shells. Together, our results indicate that the intact α-carboxysome shell can maintain a pH gradient between interior and external environments, while it is still permeable to protons to some degree. The high cytoplasmic HCO_3_^–^ concertation (>10 mM) is necessary for acidification in carboxysome shells. Moreover, the saturated HCO_3_^–^ concentration level within the α-carboxysome shell was estimated to be ∼15 mM, based on the discrepancy between the HCO_3_^–^ concentrations of α-shell-CA and α-shell. Mechanistic insights into the physiochemical conditions and regulation inside the protein shells are crucial for a better understanding of the assembly of native protein cages and catalytic performance of cargo enzymes,^37,61,68,69^ and will aid in the rational design and engineering of new protein-based nanocontainers. The developed technique may empower our analytical toolbox and provide a framework for studying native carboxysomes, diverse bacterial microcompartments, and self-assembling systems in the native context.

## Supporting Information Available

Expression and isolation of α-carboxysome shells encapsulating pH-sensitive pHluorin2 (Figure S1); *in vitro* fluorescence characterization of pHluorin2-S2C at different buffer pH 5.0–8.5 (Figure S2); fluorescence excitation spectra of free pHluorin2, α-shell-pHluorin2, and α-shell-CA-pHluorin2 expressed in *E. coli* (Figure S3); changes in the internal pH under trypsin treatment (Figure S4); changes in the buffer pH of free pHluorin2 in 20 mM NaHCO_3_ (Figure S5); gene sequence of pHluorin2-S2C (Table S1); sequences of oligonucleotide primers (Table S2) (PDF)

## Notes

A patent (PCT/GB2021/052574) has been granted for the development and use of the α-carboxysome shell.

## Abbreviations

CA: carbonic anhydrase
Rubisco: ribulose-1,5-bisphosphate carboxylase/oxygenase
GFP: green fluorescent protein
EM: electron microscopy
