# Supplementary material for: Probing the Internal pH and Permeability of a Carboxysome Shell
Source: Biomacromolecules. 2022 Sep 2;23(10):4339–48. doi: 10.1021/acs.biomac.2c00781 (PMC9554877; doi:10.1021/acs.biomac.2c00781)
Supplement: Supplementary file 1 — bm2c00781_si_001.pdf [file bm2c00781_si_001.pdf]

Supporting Information

Probing the internal pH and permeability of a carboxysome shell

Jiafeng Huang[1,2], Qiuyao Jiang[1,3], Mengru Yang[1], Gregory F. Dykes[1], Samantha L. Weetman[1], Wei Xin[3,4], Hai-Lun He[2], Lu-Ning Liu[1,5*]*

[1] Institute of Systems, Molecular and Integrative Biology, University of Liverpool, Crown Street, Liverpool L69 7ZB, United Kingdom

[2] School of Life Sciences, Central South University, Changsha 410017, China

[3] Department of Central Laboratory, Shandong Provincial Hospital Affiliated to Shandong First Medical University, Jinan 250021, China

[4] Medical Science and Technology Innovation Center, Shandong First Medical University & Shandong Academy of Medical Sciences, Jinan 271000, China

[5] College of Marine Life Sciences, and Frontiers Science Center for Deep Ocean Multispheres and Earth System, Ocean University of China, Qingdao 266003, China

* Correspondence: luning.liu@liverpool.ac.uk (L.-N.L.), helenhe@csu.edu.cn (H.-L.H.)

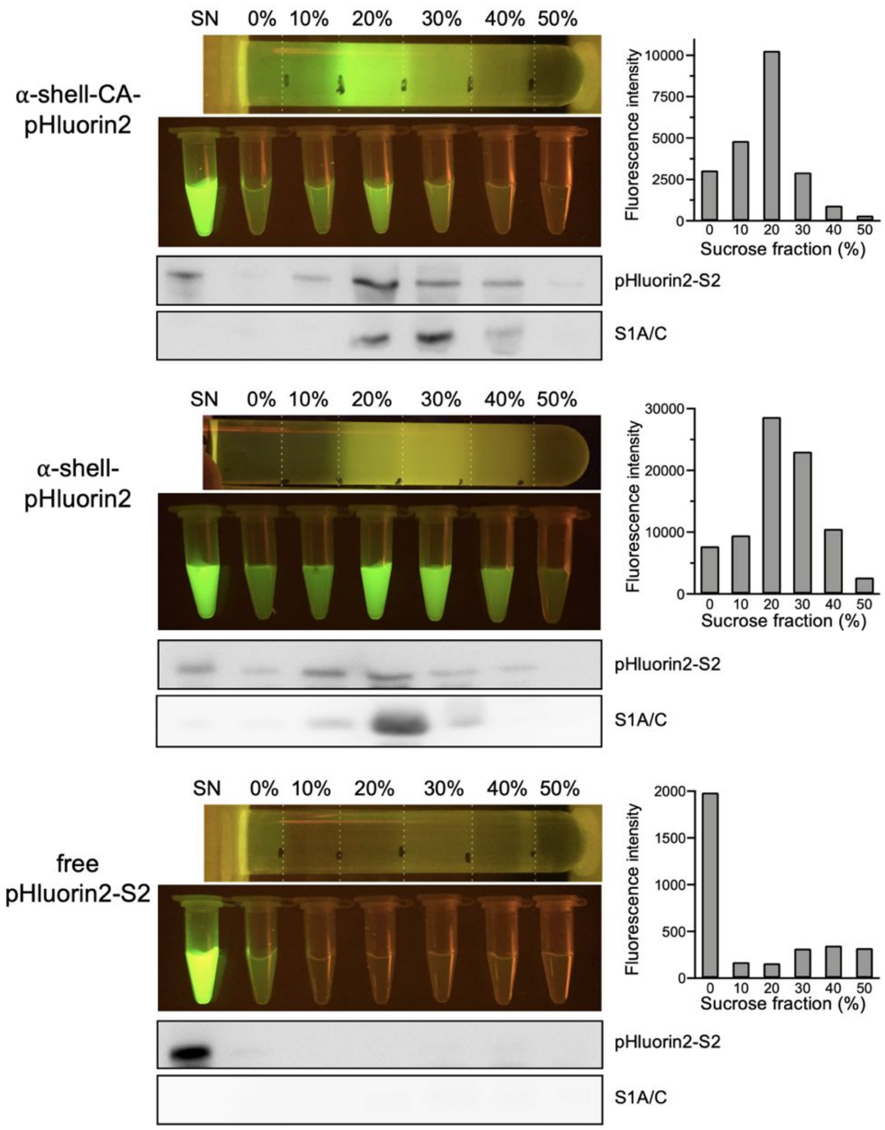

Figure S1. Expression and isolation of α-carboxysome shells encapsulating pH-sensitive pHluorin2. Isolation of α-shell-CA-pHluorin2, α-shell-pHluorin2, and free pHluorin2-S2C expressed in *E. coli* using step sucrose gradient ultracentrifugation. Fluorescence detection (left) and fluorescence intensities (right) revealed the enrichment of α-shell-CA-pHluorin2 and α-shell-pHluorin2 in 20-30% fractions, indicating the encapsulation of pHluorin2 within both the α-shell-CA and α-shell synthetic shells in comparison with free pHluorin2 at the supernatant (SN). Immunoblot analysis of α-shell-CA-pHluorin2, α-shell-pHluorin2, and free pHluorin2-S2C samples in each sucrose fraction (SN, 0-50%) using an anti-GFP antibody (for pHluorin2) and an anti-CsoS1 antibody (for CsoS1A/C), confirming the encapsulation of pHluorin2 within both the α-shell-CA and α-shell synthetic shells.

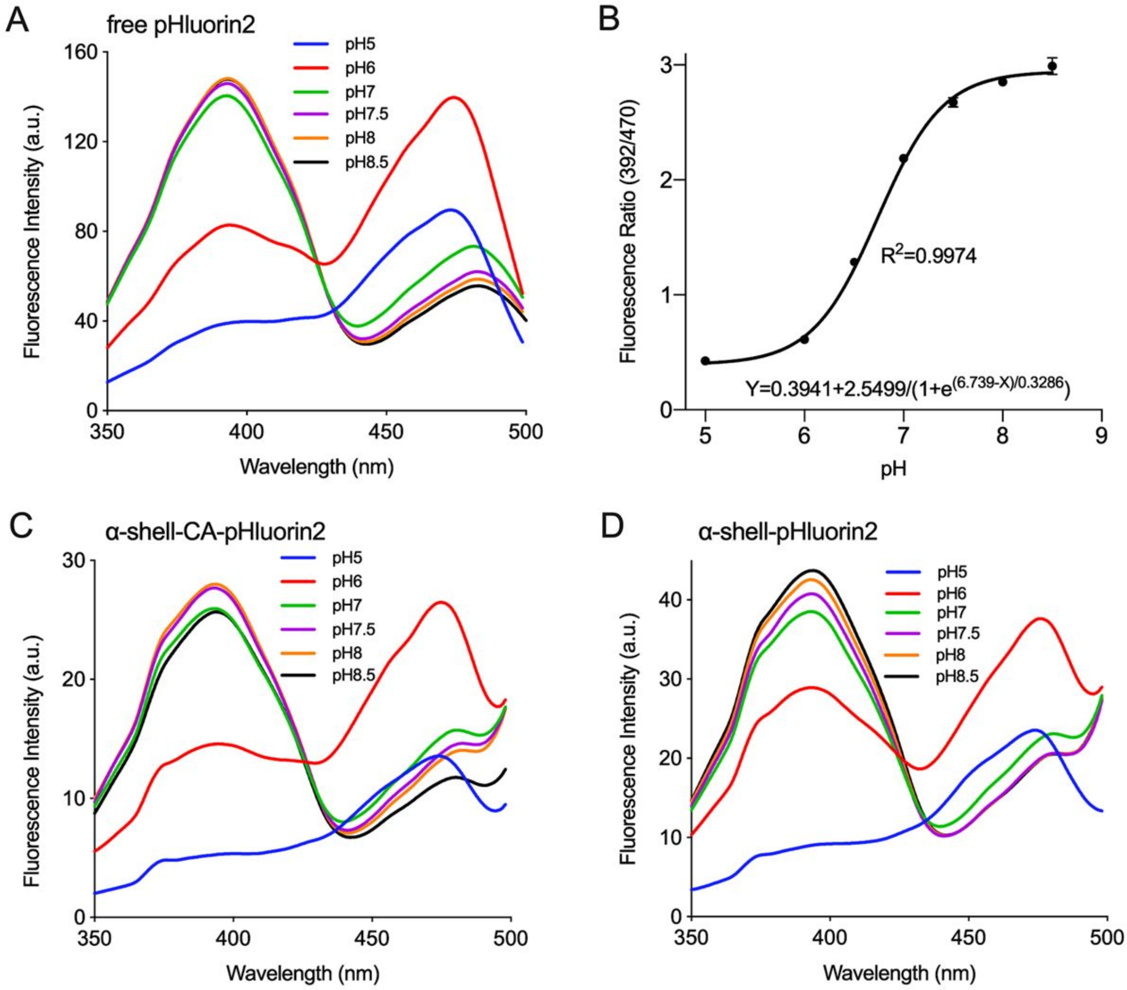

Figure S2. ***In vitro*** **fluorescence characterization of pHluorin2-S2C at different buffer pH in the range of 5.0-8.5. (A)** Fluorescence excitation of free pHluorin2-S2C at the buffer pH 5.0-8.5, as outlined in Materials and Methods. **(B)** Correlation between the free pHluorin2 fluorescence 392/470 nm ratio and buffer pH. The calibration curve was fitted by the Boltzmann sigmoid best-fitting model [$Y = 0.3941 + 2.5499/(1 + e^{(6.739 - X)/0.3286})$], and was used to determine the interior pH of carboxysome shells in different buffers. Data are representative of three independent experiments and analyzed by one way analysis of variance (ANOVA). **(C)** Fluorescence excitation of pHluorin2-S2C encapsulated within α-shell-CA at pH 5.0-8.5. **(D)** Fluorescence excitation of pHluorin2-S2C encapsulated within α-shell at pH 5.0-8.5.

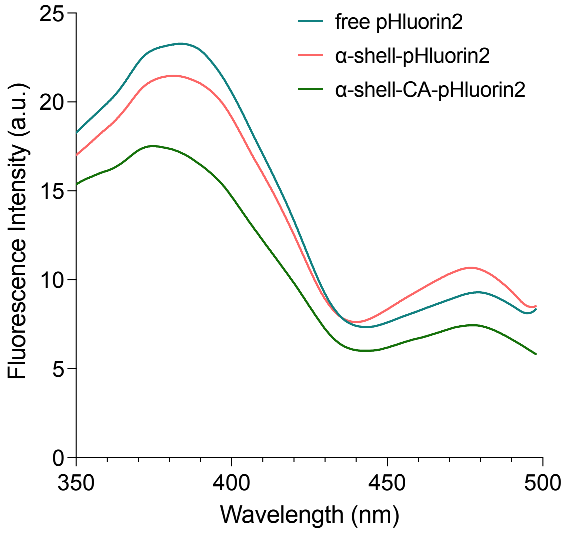

Figure S3. Fluorescence excitation spectra of free pHluorin2, α-shell-pHluorin2, and α-shell-CA-pHluorin2 with the emission at 508 nm, to determine *in situ* cytoplasmic pH and the internal pH of α-shell and α-shell-CA in *E. coli*. See also Figure 2D. n = 3 independent biological replicates.

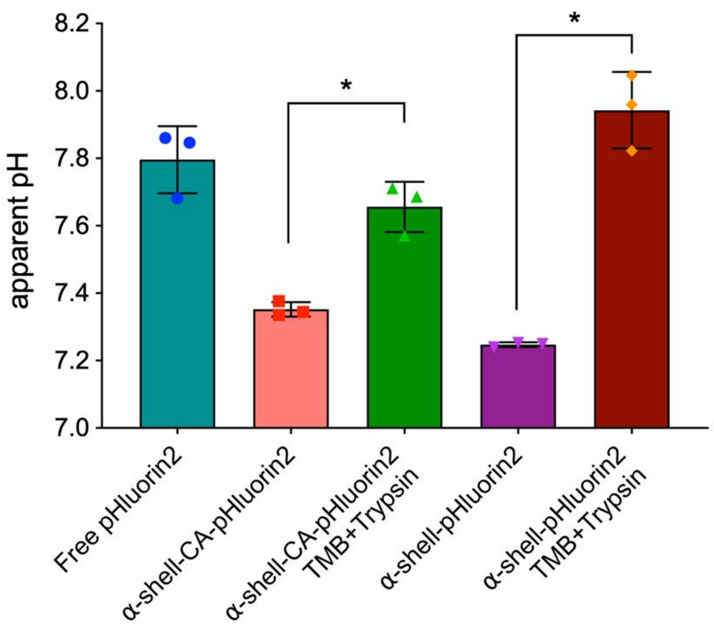

Figure S4. Structural integrity of the carboxysome shells ensures the pH gradient between internal and external environments. Trypsin digestion can disrupt the shell barrier structure and modulate shell permeability. Consequently, the interior pH of α-shell-CA and α-shell becomes closer to the TMB buffer pH. Data are representative of three independent experiments and analyzed by Student's t-test. *, $p < 0.05$.

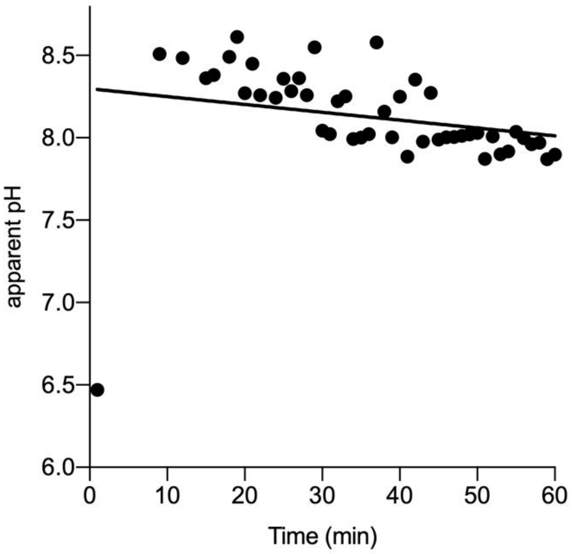

Figure S5. Dynamic changes in the buffer pH indicated by the fluorescence 392/470 nm ratios of free pHluorin2 incubated in 20 mM $NaHCO_3$ (in ddH_2O, pH 8.6). See also Figure 4.

Table S1. pHluorin2-S2C sequence and annotation. *pHluotin2* in green, *S2C* in brown, overlapping ends underlined.

GTTTAACTTTAAGAAGGAGATATACAATGGTGAGCAAGGGCGAGGAGCTGTTCACCGGGGTGGTGCCCATCCTG
GTCGAGCTGGACGGCGACGTAAACGGCCACAAGTTCAGCGTGTCCGGCGAGGGCGAGGGCGATGCCACCTACGG
CAAGCTGACCCTGAAGTTCATCTGCACCACCGGCAAGCTGCCCGTGCCCTGGCCCACCCTCGTGACCACCCTGA
GCTACGGCGTGCAGTGCTTCAGCCGCTACCCCGACCACATGAAGCAGCACGACTTCTTCAAGTCCGCCATGCCC
GAAGGCTACGTCCAGGAGCGCACCATCTTCTTCAAGGACGACGGCAACTACAAGACCCGCGCCGAGGTGAAGTT
CGAGGGCGACACCCTGGTGAACCGCATCGAGCTGAAGGGCATCGACTTCAAGGAGGACGGCAACATCCTGGGGC
ACAAGCTGGAGTACAACTACAACGAGCACCTGGTGTACATCATGGCCGACAAGCAGAAGAACGGCACCAAGGCC
ATCTTCCAGGTGCACCACAACATCGAGGACGGCAGCGTGCAGCTCGCCGACCACTACCAGCAGAACACCCCCAT
CGGCGACGGCCCCGTGCTGCTGCCCGACAACCACTACCTGCACACCCAGTCCGCCCTGAGCAAAGACCCCAACG
AGAAGCGCGATCACATGGTCCTGCTGGAGTTCGTGACCGCCGCCGGGATCACTCACGGCATGGACGAGCTGTAC
AAGGAAGCTCAATCAACTGAACAATCATTGACCTGTGAAGGACAAATTATTAGCGGCACTTCAGTTGACGCCAG
TGATTTGGTCACAGGAAATGAAATCGGTGAACAGCAACTCATCAGCGGTGACGCCTATGTTGGCGCGCAGCAGA
CAGGTTGCCTTCCCACTAGTCCACGCTTCAACCAAACTGGCAATGTTCAGTCAATGGGTTTTAAGAACACCAAT
CAGCCAGAACAAAACTTTGCACCAGGTGAAGTAATGCCTACTGACTTTAGTATTCAAACCCCAGCTCGCTCGGC
TCAGAATCGCATTACAGGTAACGACATTGCGCCCTCAGGTCGCATTACAGGCCCTGGTATGCTGGCAACCGGCT
TGATTACAGGAACCCCCGAATTCAGGCACGCTGCGCGCGAGTTGGTTGGTTCTCCACAACCCATGGCAATGGCC
ATGGCCAACCGTAATAAGGCTGCTCAAGCACCTGTTGTGCAGCCAGAAGTGGTTGCAACTCAGGAAAAGCCTGA
GTTGGTATGTGCACCAAGAAGCGATCAAATGGATCGTGTGAGTGGCGAAGGCAAAGAACGTTGCCACATCACTG
GCGATGACTGGTCAGTTAACAAGCACATCACCGGTACAGCCGGTCAATGGGCGAGTGGTCGCAACCCTTCCATG
CGCGGTAATGCGCGTGTGGTCGAAACCAGCGCGTTTGCCAATCGCAATGTGCCAAAACCTGAAAAGCCGGGCTC
CAAGATCACGGGCAGTAGTGGTAATGACACCCAAGGTAGTCTGATCACTTACTCCGGCGGCGCGCGCGGTTGAG
CACTTATTCAGGCGTAGCAAC

Table S2. Oligonucleotide primers used in this paper

Primer	Sequence (5'-3')
S2(pBAD)-Fw	GGCTAACAGGAGGAATTAACATGGGGTCAAACATGCCTTC
S1D(pBAD)-Rv	TGTTCTACGTAAGCTTCGTTAGAACCCTTCAGCGCGAC
CAKO(pBAD)-Fw	TTAAGTAAAGTGTAACGATCATGAAAATCATGCAAGTTGA
CAKO(pBAD)-Rv	TCAACTTGCATGATTTTCATGATCGTTACACTTTACTTAA
pHluorin2(pBAD33)-Fw	GTTTAACTTTAAGAAGGAGATATACAATGGTGAGCAAGGGCGAGGA
S2_pHluorin2-Rv	GATTGTTCAGTTGATTGAGCTTCCTTGTACAGCTCGTCCATGC
S2-Fw	GAAGCTCAATCAACTGAACAATC
S2(pBAD33)-Rv	GTTGCTACGCCTGAATAAGTGCTCAACCGCGCGCGCCGCC
